# Characterization of human platelet binding of recombinant T cell receptor ligand

**DOI:** 10.1186/1742-2094-7-75

**Published:** 2010-11-08

**Authors:** Asako Itakura, Joseph E Aslan, Sushmita Sinha, Tara C White-Adams, Ishan A Patel, Roberto Meza-Romero, Arthur A Vandenbark, Gregory G Burrows, Halina Offner, Owen JT McCarty

**Affiliations:** 1 Department of Cell and Developmental Biology, Oregon Health & Science University, Portland, USA; 2 Department of Biomedical Engineering, Oregon Health & Science University, Portland, USA; 3 Department of Neurology, Oregon Health & Science University, Portland, USA; 4 Department of Biochemistry and Molecular Biology, Oregon Health & Science University, Portland, USA; 5 Anesthesiology and Perioperative Medicine, Oregon Health & Science University, Portland, USA; 6 Neuroimmunology Research, Veterans Affairs Medical Center, Portland, USA; 7 Department of Pediatrics, University of Colorado Denver, Aurora, USA

## Abstract

**Background:**

Recombinant T cell receptor ligands (RTLs) are bio-engineered molecules that may serve as novel therapeutic agents for the treatment of neuroinflammatory conditions such as multiple sclerosis (MS). RTLs contain membrane distal α1 plus β1 domains of class II major histocompatibility complex linked covalently to specific peptides that can be used to regulate T cell responses and inhibit experimental autoimmune encephalomyelitis (EAE). The mechanisms by which RTLs impede local recruitment and retention of inflammatory cells in the CNS, however, are not completely understood.

**Methods:**

We have recently shown that RTLs bind strongly to B cells, macrophages, and dendritic cells, but not to T cells, in an antigenic-independent manner, raising the question whether peripheral blood cells express a distinct RTL-receptor. Our study was designed to characterize the molecular mechanisms by which RTLs bind human blood platelets, and the ability of RTL to modulate platelet function.

**Results:**

Our data demonstrate that human blood platelets support binding of RTL. Immobilized RTL initiated platelet intracellular calcium mobilization and lamellipodia formation through a pathway dependent upon Src and PI3 kinases signaling. The presence of RTL in solution reduced platelet aggregation by collagen, while treatment of whole blood with RTL prolonged occlusive thrombus formation on collagen.

**Conclusions:**

Platelets, well-known regulators of hemostasis and thrombosis, have been implicated in playing a major role in inflammation and immunity. This study provides the first evidence that blood platelets express a functional RTL-receptor with a putative role in modulating pathways of neuroinflammation.

## Background

Recombinant T cell receptor ligands (RTLs) represent a novel, bio-engineered therapeutic drugs for T cell-mediated autoimmune diseases. RTL molecules consist of the membrane distal α1 plus β1 domains of class II major histocompatibility complex molecules and contain covalently linked peptide antigen to induce immunosuppression by crosslinking to T cell receptor (TCR) in the absence of co-stimulatory signals[1]. By inhibiting autoreactive T cell responses, RTLs have been shown to reverse the clinical and histological signs in experimental autoimmune encephalomyelitis (EAE)[2], although the molecular mechanisms by which RTLs inhibit T cell proliferation and cytokine secretion are still poorly defined. While RTLs displayed preferential binding to murine antigen presenting cells (APCs) such as B cells, macrophages and dendritic cells, but not to T cells[3], the binding targets expressed on APCs are currently unknown.

Blood platelets are classically considered as key regulators of hemostasis. Platelets, however, are also emerging as modulators in immune responses as well as in the etiology of neuropathologies[4]. Platelets possess a wide array of adhesion receptors and secretory products, consisting of chemokines and cytokines[5]. It has been proposed that platelets partner with leukocytes to amplify the immune response at sites of tissue repair or inflammation[6,7]. Along these lines, in a murine model of pulmonary acute lung injury, blockade of platelet-derived thromboxane reversed disease progression, while pharmacological inhibition of platelet-leukocyte interactions with P-selectin antibodies reduced pulmonary inflammation[8,9]. Accordingly, the presence of platelet-specific markers such as P-selectin and platelet microparticles in MS patients[10,11] suggests that platelets may contribute to the pathophysiology of MS[4,12]. Thus, pharmacological regulation of platelet function may represent a potential therapeutic strategy for the treatment of neurovascular inflammation.

## Materials and methods

### Reagents

Plasma-derived fibrinogen was from Enzyme Research Laboratories, Inc. (South Bend, IN, USA). RTL1000 and RTL551 was synthesized as previously described[13]. Anti-factor XI mAb was generated and purified as described[14]. All other reagents were from Sigma-Aldrich, Inc. (St. Louis, MO, USA) or previously named sources[15].

### Preparation of purified platelets

Human venous blood was collected from healthy volunteers into sodium citrate (final concentration 0.38% vol/vol) and acid/citrate/dextrose (ACD, 10% vol/vol) to purify the platelets as previously described[15]. Briefly, platelet-rich plasma (PRP) was prepared by centrifugation of whole blood at 200 g for 20 minutes. The platelets were isolated from PRP by centrifugation at 1000 g for 10 minutes in the presence of prostacyclin (0.1 μg/ml). After centrifugation, purified human platelets were resuspended in modified Tyrode's buffer (129 mM NaCl, 0.34 mM Na_2_HPO_4_, 2.9 mM KCl, 12 mM NaHCO_3_, 20 mM HEPES, 5 mM glucose, 1 mM MgCl_2_; pH 7.3). Mouse platelets were purified as previously described[16].

### Static adhesion assays

Glass coverslips were incubated with a 50 μg/ml solution of RTL1000 or fibrinogen for 1 hour at room temperature. Surfaces were then blocked with denatured fatty acid-free bovine serum albumin (BSA, 5 mg/ml) for 1 hour and washed with phosphate-buffered saline (PBS). Purified human or mouse platelets (2 × 10^7^/ml) were incubated on the protein-coated coverslips at 37°C for 45 minutes. Platelet spreading was imaged using Kohler illuminated Nomarski differential interference contrast (DIC) optics with a Zeiss 63× oil immersion 1.40 NA plan-apochromat lens on a Zeiss Axiovert 200 m microscope (Carl Zeiss). Images were collected and processed using Stallion 4.0 (Intelligent Imaging Innovations Inc, Denver, CO). The degree of platelet adhesion and surface area of bound platelets was quantified using Image J software as previously described[16].

### Fluorescent binding assay

RTL1000 and RTL551 was processed using Zeba™ Desalt Spin Column (Thermo Fisher Scientific, Waltham, MA) for buffer-exchange, retained in 50 mM HEPES according to the manufacturer's instruction and labeled with Alexa Fluor^® ^488 using Alexa Fluor^® ^488 labeling kit (Invitrogen, Carlsbad, CA). Labeled RTL1000 and RTL551 (RTL1000-Alexa488 and RTL551-Alexa488, respectively) were processed using the spin column and recovered in 20 mM Tris (pH 8.4). The yield of the fluorescent protein was confirmed by measuring OD_480 _and OD_290_. Purified human or mouse platelets were incubated at 37°C for 30 min on fibrinogen-coated glass coverslips followed by washing with PBS. RTL1000-Alexa488 or RTL551-Alexa488 (20 μg/ml) in BSA (1 mg/ml) solution was then loaded over the adherent human or mouse platelets, respectively, and incubated at 37°C for 30 min. Fluorescence was recorded with a Zeiss Axiovert inverted fluorescent microscope.

### Single platelet Ca^2+ ^measurements

Platelets were loaded with 15 μM Oregon Green BAPTA1-AM (Molecular Probes, Eugene, OR) as previously described[17]. Loaded platelets were allowed to sediment onto RTL- or fibrinogen-coated coverslips over a period of 30 min at 37°C. Fluorescent intensities in single platelets were recorded and analyzed using a custom Matlab program.

### Western blotting and phosphorylation studies

Purified human platelets were incubated for 45 minutes on protein-coated 24 well plates and lysed with an ice-cold lysis buffer (20 mM Tris, 300 mM NaCl, 2 mM EGTA, 2 mM EDTA, 2% NP-40 pH 7.5, containing Roche Complete Protease Inhibitor Cocktail). Lysates were separated by SDS-PAGE and transferred onto PVDF membranes. The membranes were blocked with 5% BSA for 1 hour prior to incubation with primary antibodies overnight at 4°C. Following washing with TBS-T, membranes were incubated with the appropriate secondary antibodies. Subsequent immunoblotting was carried out as previously described[18].

### Platelet aggregation and clotting time measurements

Platelet aggregation was assayed using 300 μl of washed platelets (2 × 10^8^/ml) in a Chrono-Log aggregometer (Chrono-Log, Havertown, PA) with continuous stirring at 1200 rpm at 37°C. Activated partial thromboplastin time (aPTT) of pooled human platelet-poor plasma (PPP) and recalcification time of PPP were measured with a KC4 Coagulation Analyzer (Trinity Biotech PLC, Co Wicklow, Ireland). Samples were pre-treated with either vehicle, RTL1000 (10 μg/ml), an anti-FXI mAb (14E11, 20 μg/ml) or tissue factor (TF, 1 pM) for 3 min prior to the addition of 16.6 mM CaCl_2 _for recalcification times or aPTT reagent (Helena Laboratories, Beaumont, TX) followed by 6.6 mM CaCl_2 _for aPTT tests. In each test, clotting time was measured following the addition of CaCl_2 _as previously described[14].

### Capillary occlusion assay

Capillary tubes were coated with fibrillar collagen, aligned vertically and connected to a reservoir as previously described[14]. Sodium-citrate (0.38% w/v) anticoagulated whole blood was sequentially supplemented with 7.5 mM Ca^2+ ^and 3.75 mM Mg^2+^. Capillary flow was driven by the force of gravity, and the height of the sample reservoir was regulated in order to produce an initial shear rate of 300 s^-1 ^as previously described[19].

### Analysis of data

Data are shown as means ± SEM. Statistical significance of differences between means was determined by ANOVA. If means were shown to be significantly different, multiple comparisons were performed by the Tukey test. Probability values of *P *< 0.05 were selected to be statistically significant.

## Results

### Characterization of RTL as a ligand for platelets

**Figure 1:**
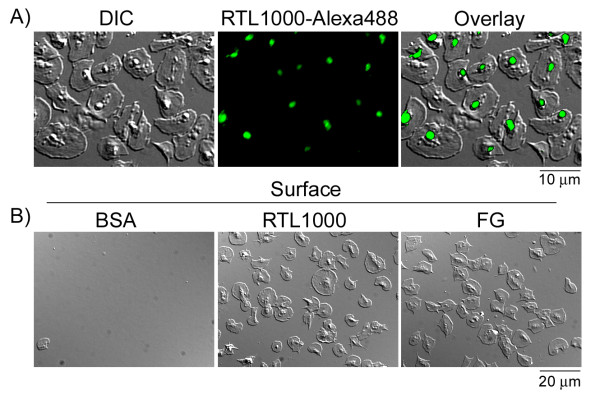
**Characterization of RTL1000-human platelet binding**. A) Immobilized human platelets (2 × 10^7^/ml) were incubated with fluorescently-labeled RTL1000 (RTL1000-Alexa488, 20 μg/ml) for 30 min at 37°C. Cells were imaged using differential interference contrast (DIC) and fluorescent microscopy. B) Washed human platelets (2 × 10^7^/ml) were pipetted onto surfaces coated with RTL1000 (50 μg/ml) or fibrinogen (FG; 50 μg/ml) for 45 min at 37°C.

To determine the ability of human blood platelets to support RTL1000 binding, purified platelets were immobilized on a surface of fibrinogen prior to exposure to fluorescently-labeled RTL1000. RTL1000 bound to the human platelet surface (Figure 1A), demonstrating that a receptor for RTL may be present on the surface of human platelets. In an attempt to identify a potential RTL-receptor on platelets, washed human platelets were incubated over RTL-coated glass coverslips, and platelet adhesion and spreading was monitored with Nomarski differential interference contrast (DIC) microscopy. Our data show that RTL1000 supported human platelet surface adhesion and lamellipodia formation (Figure 1B). The degree of adhesion observed was similar to that of platelet adhesion to an immobilized surface of fibrinogen, which supports platelet adhesion through the α_IIb_β_3 _integrin. Minimal platelet adhesion was observed on BSA-coated coverslips (Figure 1B).

**Figure 2:**
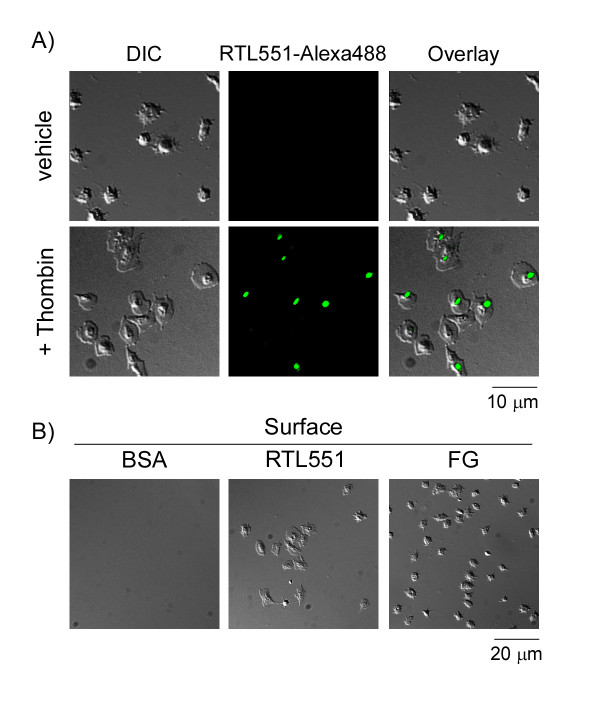
**Characterization of RTL551-mouse platelet binding**. A) Immobilized mouse platelets (2 × 10^7^/ml) were incubated with fluorescently-labeled RTL551 (RTL551-Alexa488, 20 μg/ml) for 30 min at 37°C. In selected experiments, mouse platelets were stimulated with thrombin (1 U/ml). Cells were imaged using differential interference contrast (DIC) and fluorescent microscopy. B) Washed mouse platelets (2 × 10^7^/ml) were pipetted onto surfaces coated with RTL551 (50 μg/ml) or fibrinogen (FG; 50 μg/ml) for 45 min at 37°C.

A parallel series of experiments was performed with purified mouse platelets. RTL551 contains the peptide derived from mouse myelin oligodendrocyte glycoprotein (MOG_33-35_)[3]. Our data demonstrate that fluorescently-labeled RTL551 bound to the mouse platelet surface, yet only following stimulation of mouse platelets with the G protein-coupled agonist, thrombin (Figure 2A). Immobilized RTL551 supported mouse platelet surface adhesion and lamellipodia formation (Figure 2B). Consistent with previous reports, mouse platelets exhibit only partial spreading on a fibrinogen surface (Figure 2B).

### RTL supports platelet actin cytoskeletal reorganization and Ca^2+ ^mobilization

**Figure 3:**
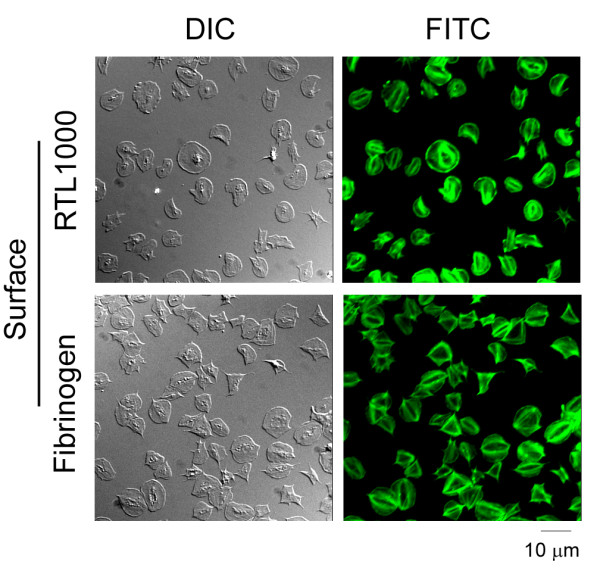
**Platelet cytoskeletal reorganization on RTL1000 surfaces. **Platelets bound to RTL1000 or fibrinogen surfaces were fixed, permeabilized and stained for F-actin using FITC-conjugated phalloidin. Representative DIC and fluorescent microscopy images are shown.

**Figure 4:**
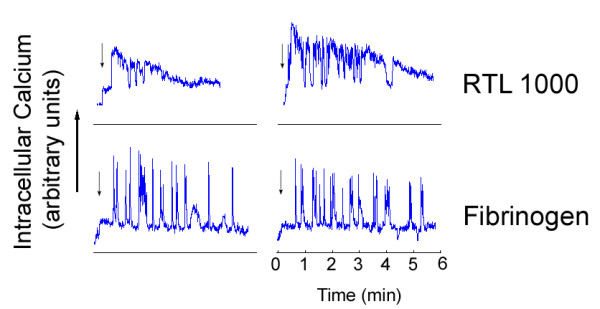
**Real-time imaging of intracellular Ca**^**2+ **^**in platelets**. Platelets were loaded with the calcium reporter dye Oregon Green BAPTA 1-AM and imaged upon contact with RTL1000 or fibrinogen-coated surfaces. Graphs show the observed fluorescent intensity (arbitrary units) of a single platelet over the period of 6 minutes. The arrows indicate the platelets' arrival at the region of interest.

Upon activation, platelets rapidly mobilize intracellular stores of calcium and assemble actin-rich structures such as filopodia, lamellipodia and stress fibers. Fluorescent labeling of the actin cytoskeleton showed the formation of stress fibers in the human platelets on immobilized RTL1000 (Figure 3). Real-time imaging of platelets loaded with the calcium-reporter dye, Oregon Green BAPTA 1-AM, revealed that platelets generated a rapid burst of oscillating intracellular Ca^2+ ^following binding to RTL1000, which subsequently declined over a period of 3-10 min (Figure 4). In contrast, a rhythmic series of Ca^2+ ^spikes were observed on fibrinogen (Figure 4).

### Characterization of the platelet signaling cascade downstream of RTL

**Figure 5:**
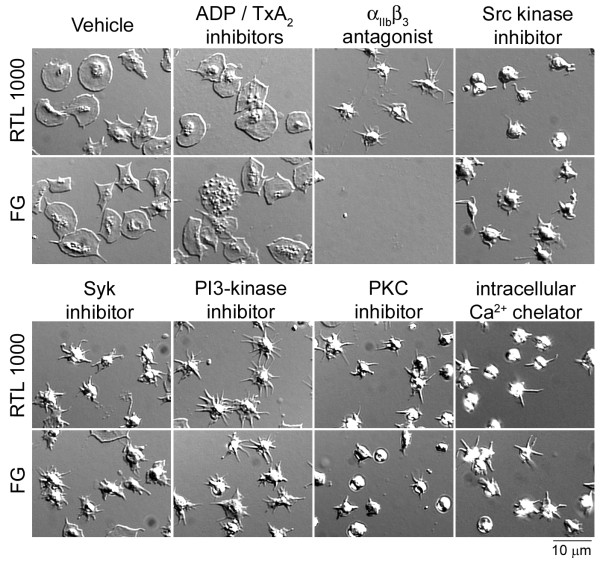
**Characterization of platelet adhesion and spreading on RTL1000**. Platelets were pipetted onto glass surfaces coated with RTL1000 or fibrinogen (FG) for 45 min at 37°C and imaged using DIC microscopy. In selected experiments, platelets were pretreated with vehicle, the ADP scavenger apyrase (2 U/ml) and cyclooxygenase inhibitor indomethacin (10 μM), the integrin α_IIb_β_3 _antagonist eptifibatide (20 μg/ml), inhibitors to Src family kinases (PP2, 20 μM), Syk kinase (BAY 61-3606, 20 μM), PI3 kinases (wortmannin, 100 nM), protein kinase C (R0 31-8220, 20 μM) or an intracellular Ca^2+ ^chelator (BAPTA-AM, 10 μM).

We next aimed to determine the intracellular mechanisms that mediate human platelet spreading on RTL1000. Addition of the ADP-scavenger apyrase in combination with the cyclooxygenase inhibitor indomethacin had no effect on platelet spreading on RTL1000 (Table 1). In contrast, treatment of platelets with inhibitors to Src kinases (PP2, 20 μM), Syk kinase (BAY 61-3606, 20 μM), PI3 kinases (wortmannin, 100 nM), protein kinase C (R0 31-8220, 20 μM) or an intracellular Ca^2+ ^chelator (BAPTA-AM, 10 μM) abrogated platelet lamellipodia formation on RTL1000-coated surfaces. Platelet lamellipodia formation on RTL1000 was also inhibited in the presence of the α_IIb_β_3 _receptor antagonist, eptifibatide, whereas adhesion to fibrinogen was eliminated in the presence of eptifibatide (Figure 5 and Table 1)

**Table 1:** Effects of inhibitors and antagonists on platelet surface area

	Platelet adhesion (cells/mm^2 ^× 10^2^)		Surface area (μm^2^)	
	
Inhibitor/antagonists	RTL1000	fibrinogen	RTL1000	fibrinogen
vehicle	44.9 ± 1.1	52.8 ± 1.6	35.5 ± 3.1	31.8 ± 1.2

ADP-scavenger/cyclooxygenase inhibitor	33.4 ± 0.5	34.3 ± 0.7	34.5 ± 3.9	32.1 ± 2.2

α_IIb_β_3 _antagonist	26.4 ± 0.8	7.9 ± 1.5	17.6 ± 1.8	16.7 ± 2.3

Src kinase inhibitor	26.4 ± 0.8	48.4 ± 1.9	23.7 ± 2.0	20.0 ± 1.5

Syk kinase inhibitor	36.1 ± 0.5	44.0 ± 0.4	19.5 ± 1.9	25.0 ± 1.4

PI3 kinase inhibitor	29.0 ± 0.5	30.8 ± 0.9	26.3 ± 1.7	17.3 ± 1.4

protein kinase C inhibitor	36.7 ± 0.5	53.7 ± 1.7	19.4 ± 2.3	27.3 ± 1.6

intracellular Ca^2+ ^chelator	11.4 ± 1.1	13.2 ± 1.2	15.5 ± 2.1	17.2 ± 1.4

Purified human platelets (2 × 10^7^/ml) were placed on RTL1000- or fibrinogen-coated coverslips for 45 min at 37°C with an ADP-scavenger and cyclooxygenase inhibitor (apyrase/indometacin, 2 U/ml/10 μM), the α_IIb_β_3 _receptor antagonist (eptifibatide, 20 μg/ml), inhibitors to Src kinases (PP2, 20 μM), Syk kinase (BAY 61-3606, 20 μM), PI3 kinase (wortmannin, 100 nM), protein kinase C (R0 31-8220, 20 μM) or an intracellular Ca^2+ ^chelator (BAPTA-AM, 10 μM). Values are reported as mean ± SEM of 50-300 cells

**Figure 6:**
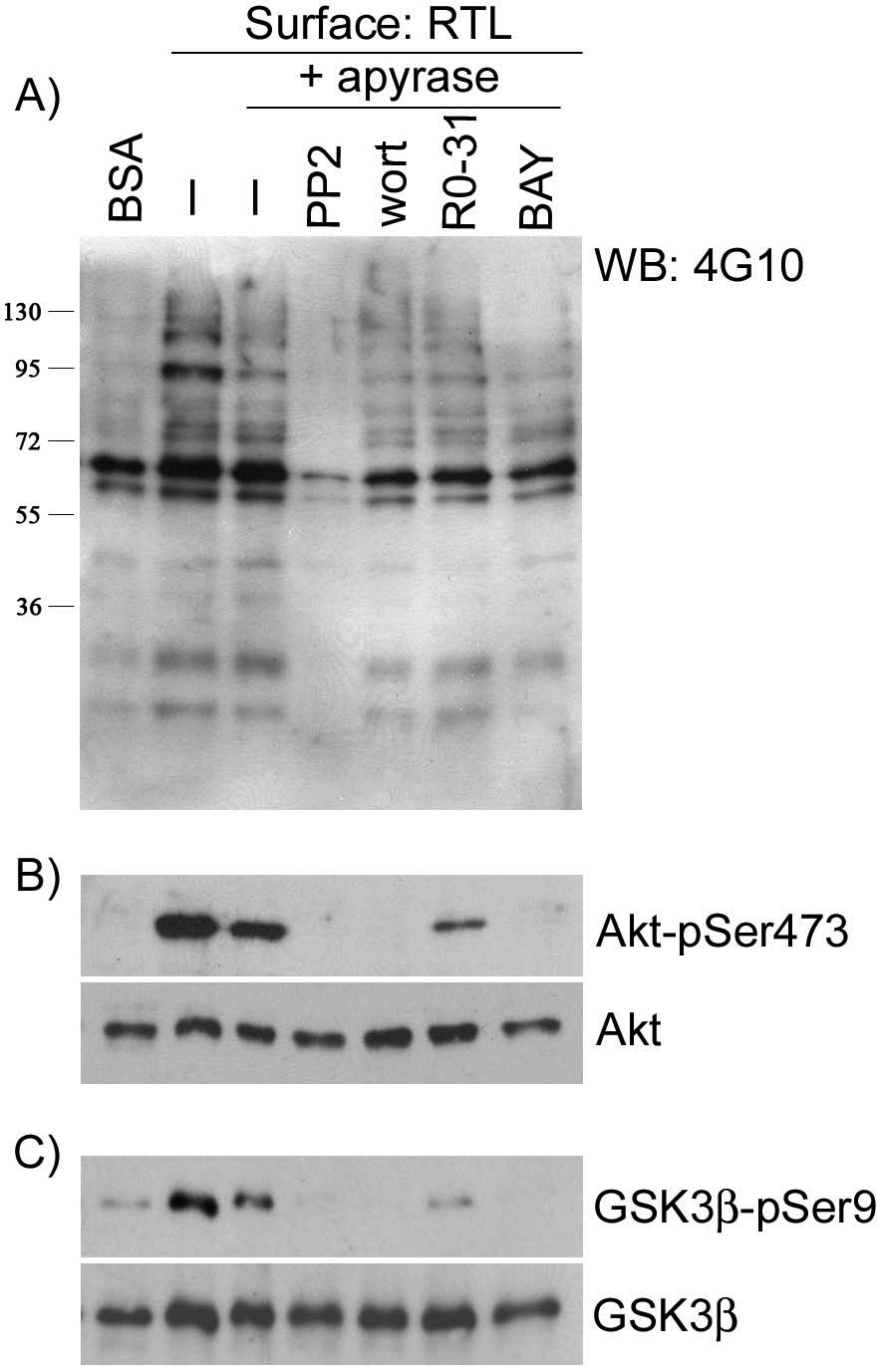
**Characterization of phosphorylation in platelets downstream of RTL1000**. Human washed platelets were exposed to BSA, RTL1000, or fibrinogen (FG) surfaces for 45 min at 37°C. Platelets adherent to RTL1000 or in suspension over BSA were lysed in ice-cold lysis buffer. Platelet lysates were separated by SDS-PAGE and immunoblotted for phosphorylation using A) an anti-phosphotyrosine antibody (4G10), B) anti-Akt antibody, or C) anti-GSK3β antibody. In selected experiments, platelets were pretreated with vehicle or the ADP scavenger apyrase (2 U/ml) in the absence or presence of inhibitors to Src kinases (PP2, 20 μM), PI3 kinases (wortmannin, 100 nM), protein kinase C (R0 31-8220, 20 μM), or Syk kinase (BAY 61-3606, 20 μM).

We next determined whether the interaction of RTL1000 with human platelets led to the activation of signal transduction pathways with known roles in platelet adhesion and spreading. Platelet binding to RTL1000 was associated with an increase in total tyrosine phosphorylation levels as compared to platelets under control conditions (platelets in solution over BSA) (Figure 6A). The addition of the ADP-scavenger apyrase reduced the degree of tyrosine phosphorylation in whole cell lysates as well as the phosphorylation of Akt and of GSK3β (Figure 6A, B and 6C). The Src family kinase inhibitor, PP2, the PI3 kinase inhibitor, wortmannin, or the Syk kinase inhibitor, BAY 61-3606, abrogated phosphorylation of Akt and GSK3β (Figure 6B and 6C). In contrast, phosphorylation of Akt and GSK3β was not affected by the protein kinase C inhibitor, R0 31-8220 (Figure 6B and 6C).

### Effect of RTL on platelet aggregation, coagulation and occlusive thrombus formation

**Figure 7:**
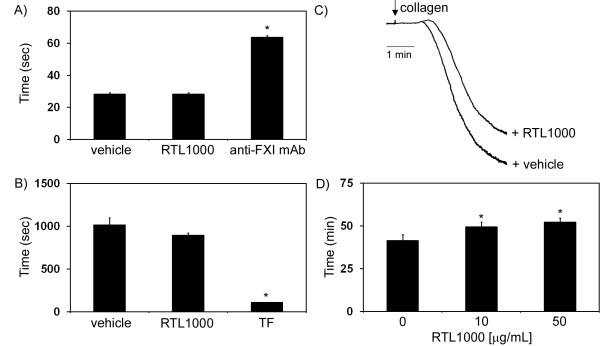
**Effect of RTL on platelet function and occlusive thrombus formation**. The effect of RTL1000 on A) aPTT or B) the clotting time of PPP in the presence of excess molar Ca^2+^. In selected experiments, plasma was pretreated with vehicle, RTL1000 (10 μg/ml), the anti-FXI mAb 14E11 (20 μg/ml) or tissue factor (TF, 1 pM). C) Washed human platelets (2 × 10^8^/ml) were pretreated with either vehicle or RTL1000 (10 μg/ml) for 2 min prior to stimulation with collagen (2 μg/ml). The extent of platelet aggregation was observed using a Chrono-Log aggregometer. D) Whole human blood in 0.38% sodium citrate was recalcified and perfused through a collagen-coated glass capillary until occlusion. Blood flow was driven by a constant pressure difference. In selected experiments, blood was pre-treated with either 10 or 50 μg/ml RTL1000. Data are reported as mean ± SEM of at least 3 experiments. * P < 0.05 compared to vehicle.

Next we determined the effect of RTL on the initiation of coagulation. RTL1000 did not affect the activated partial prothrombin time (aPTT) or the clotting time of platelet-poor plasma (PPP) initiated by the addition of excess molar Ca^2+ ^(Figure 7A and 7B, respectively). In contrast, the addition of the anti-FXI mAb, 14E11, prolonged the aPTT (Figure 7A), while the addition of tissue factor (TF, 1 pM) drastically reduced clotting time in recalcified plasma (Figure 7B).

To assess the effect of RTL on platelet function, a solution of purified human platelets was treated with RTL under constant stirring in an aggregometer. Platelet aggregation induced by the platelet agonist, collagen, was inhibited by the addition of RTL1000 (29% aggregation vs. 20% aggregation after 2 min in the presence of vehicle or RTL1000, respectively; Figure 7C). Addition of RTL1000 alone failed to initiate either platelet shape changes or aggregation (data not shown).

We next investigated the effect of RTL on occlusive thrombus formation. Recalcified blood was driven by a constant pressure gradient through collagen-coated capillaries at an initial shear rate of 300 s^-1^, and flow through the capillary was monitored until occlusion. Our data demonstrate that RTL substantially prolonged the time to occlusion (19.5% vs. 26.3% increase with respect to vehicle for 10 μg/ml RTL or 50 μg/ml RTL, respectively; Figure 7D). Taken together, these findings provide the first evidence that RTL-platelet binding may negatively regulate platelet function and prolong occlusive thrombus formation.

## Discussion

Multiple sclerosis is regarded as a prototypical disease state resultant of neuroinflammation. RTLs have been engineered to augment antigen-driven immunosuppression of autoreactive T cells by presenting TCR ligands (self-antigen plus MHC molecule) in the absence of co-stimulatory signals that are normally supplemented by antigen presenting cells (APCs)[1]. RTLs have been shown to reduce T cell-driven encephalitogenicity and both clinical and histological signs of EAE[2]. Previous studies suggested that RTLs confer a therapeutic benefit via the downregulation of T cell-mediated inflammation[20] and by modifying the permeability of the blood-brain barrier in a murine model of EAE[21] or ischemic stroke[22]. RTLs were found to decrease the levels of systemic chemokines and adhesion molecules on the CNS endothelium as well as cytokines that switch on anti-inflammatory effectors rather than increasing antibody production by B cells[21]. Although RTLs were originally thought to act as a partial agonist via TCR[23], our recent study demonstrated the inability of splenic CD3^+ ^T cells to bind RTLs. Rather, we found that RTLs bound APC populations including B cells, macrophages, and dendritic cells, in an antigenic-independent manner[3]. Perhaps RTL-armed splenic APCs suppress the transfer of EAE by antigen-stimulated T cells. Alternatively, peripheral blood cells may bind RTL through an unknown surface receptor distinct from TCR, indirectly inducing T cell tolerance against self-antigen. Our study was designed to determine whether peripheral blood cell populations, such as platelets, can bind to RTL, thus offering a level of redundancy to APCs.

Platelets, well-known regulators of hemostasis and thrombosis, have been implicated in playing an essential role in inflammation and immunity[5,7]. Activated platelets shed granules and microparticles which contain a variety of adhesive molecules and immunomodulatory factors, resulting in the localized recruitment of immune cells under shear. Abnormal platelet activation (microparticle presence, P-selectin expression, and increased circulating aggregates) has been documented in MS patients[11]. A recent gene microarray study revealed that the platelet α_IIb_β_3 _receptor was transcriptionally upregulated in MS chronic lesions[10]. Perhaps RTL may modulate the pathophysiological role that platelets play in neurodegenerative diseases such as MS.

A number of platelet ligands and pathways have been identified that negatively regulate platelet function. For instance, recent studies have shown negative regulatory roles for the platelet ligands semaphorin 3A[24], histone H1[25], thrombospondin-1[26], and low-density lipoprotein[27]. Our current study provides evidence that RTL, in addition to serving as a platelet ligand, may serve to negatively regulate agonist-induced platelet function, as evidenced by the fact that RTL down-regulated platelet aggregation to collagen. Moreover, our study provides evidence that RTL inhibits occlusive thrombus formation on collagen-coated surfaces under physiologically relevant pressure gradients.

Among various platelet surface RTL-receptors, one potential candidate we identified was CD40, a co-stimulatory molecule that regulates lymphocyte signaling upon antigen presentation. CD40 is known to contribute to the initiation, activation, and amplification of immune responses[28]. CD40-CD40 ligand (CD40L, or CD154) interactions are well described in MS, as CD40L^+ ^T cells are known to be greatly increased in MS patients[29,30]. Moreover, CD40 is expressed on platelets and APCs, but not T cells, in accord with the peripheral blood populations we have shown to bind RTL. We therefore tested the hypothesis that CD40 was the platelet receptor for RTL. Our preliminary findings demonstrated that purified platelets from CD40^-/- ^mice bind to RTL at the same level as compared to platelets from wild type mice (unpublished observations, AI and OJTM), arguing against a role for CD40 as an RTL-receptor on mouse platelets. Our future efforts will be focused on identifying the putative RTL-receptor on peripheral blood platelets. The characterization of the molecular mechanisms by which RTL binds to peripheral blood cells may better the understanding of the pharmacokinetics of RTL drug delivery as well as the further development of RTL for therapy in autoimmune diseases.

## Competing interests

Drs. Offner, Burrows, Vandenbark, and OHSU have a significant financial interest in Artielle ImmunoTherapeutics, Inc., a company that may have a commercial interest in the results of this research and technology. This potential conflict of interest has been reviewed and managed by the OHSU and VAMC Conflict of Interest in Research Committees.

## Authors' contributions

AI, JEA, SS, TCW, IAP and RMR designed and performed experiments, and drafted the manuscript. AAV and GGB participated in the study design and coordination. OJTM and HO designed and performed experiments, co-conceived of the study, and helped draft the manuscript. All authors have read and approved the final version of the manuscript.
